# The Influence of Meteorological Factors on Osteoarthritis Symptoms and Physical Activity: A Narrative Review

**DOI:** 10.15388/Amed.2026.33.1.3

**Published:** 2026-07-22

**Authors:** Karolis Strašunskas, Matas Mikalauskas, Ona Montvydaitė-Kreivaitienė

**Affiliations:** 1 Faculty of Medicine, Lithuanian University of Health Sciences, Kaunas, Lithuania; 2 Faculty of Medicine, Lithuanian University of Health Sciences, Kaunas, Lithuania; 3 Department of Rheumatology, Hospital of the Lithuanian University of Health Sciences Kaunas Clinics, Kaunas, Lithuania

**Keywords:** osteoarthritis, weather, temperature, humidity, barometric pressure, physical activity, osteoartritas, orai, temperatūra, drėgnumas, barometrinis slėgis, fizinis aktyvumas

## Abstract

**Background:**

Meteorological conditions are suspected to influence osteoarthritis (OA) symptoms, but findings across climates and populations are inconsistent. Understanding how temperature, humidity, and barometric pressure affect pain, stiffness, and physical activity is relevant for clinical counselling and study design.

**Objective:**

To synthesize recent evidence on associations between natural weather variation and OA symptomatology, including impacts on physical activity and rehabilitation participation.

**Methods:**

We searched *PubMed, ScienceDirect*, and *Google Scholar* for peer-reviewed human studies published from January 2010 to May 2024 by using terms related to osteoarthritis and meteorological factors (temperature, humidity, barometric pressure, precipitation, seasonal variation) combined with pain, stiffness, function, or physical activity. Inclusion criteria comprised clinically or radiographically confirmed OA and quantitative outcomes linked to measured meteorological exposures. Interventional artificial heat/cold studies and non-primary data were excluded. Two reviewers independently screened, selected, and extracted data with consensus resolution.

**Results:**

Temperature showed the most consistent patterns: colder and more variable conditions were linked to increased pain and stiffness, whereas warmer, stable conditions favored mobility. Humidity and barometric pressure fluctuations showed modest effects, particularly under cool and damp conditions. Precipitation, sunlight, and wind had minimal or inconsistent associations. Adverse weather was associated with a reduced outdoor activity and lower rehabilitation participation.

**Conclusions:**

Weather acts as a contextual modifier rather than a primary determinant of OA symptoms. Recognizing meteorological influences may aid patient counselling, season-sensitive rehabilitation planning, and the design of outcome studies.

## Abstract (it)

**Įvadas:**

Meteorologinės sąlygos galimai turi įtakos osteoartrito (OA) simptomams, tačiau tyrimų rezultatai, kuriuos lėmė skirtingas klimatas ir populiacijos, yra nevienodi. Suprasti orų temperatūros, santykinio drėgnumo ir barometrinio slėgio poveikį sergant OA skausmui, sąstingiui ir fiziniam aktyvumui, yra svarbu klinikiniam konsultavimui ir tyrimų planavimui.

**Tikslas:**

Apibendrinti naujausius natūralių orų pokyčių ryšio su OA simptomatika, įskaitant poveikį sergančiųjų šia liga fiziniam aktyvumui ir dalyvavimui reabilitacijoje, įrodymus.

**Metodai:**

Atlikta paieška „PubMed“, „ScienceDirect“ ir „Google Scholar“ duomenų bazėse, ieškant recenzuotų žmogaus tyrimų, publikuotų 2010 m. sausio – 2024 m. gegužės mėn. Naudoti raktažodžiai, susiję su osteoartritu ir meteorologiniais veiksniais (temperatūra, santykinis oro drėgnumas, barometrinis slėgis, krituliai, sezoniniai pokyčiai), derinami su skausmu, sąstingiu, funkcija ar fiziniu aktyvumu. Įtraukimo kriterijai: klinikinė ar radiologinė OA diagnozė ir kiekybiniai rezultatai, susiję su matuotais meteorologiniais veiksniais. Išskirti dirbtinio šilumos / šalčio intervenciniai tyrimai ir nepirminiai duomenys. Du recenzentai nepriklausomai atrinko ir susistemino duomenis.

**Rezultatai:**

Temperatūra parodė labiausiai nuoseklius dėsningumus: šaltesnės ir labiau kintančios sąlygos siejamos su padidėjusiu skausmu ir sąstingiu, o šiltesnės, stabilesnės – su geresniu judrumu. Santykinio drėgnumo ir barometrinio slėgio svyravimai turėjo menką poveikį, ypač vėsiomis ir drėgnomis sąlygomis. Krituliai, saulės šviesa ir vėjas turėjo minimalų ar nenuoseklų ryšį. Nepalankūs orai siejami su sumažėjusiu lauko aktyvumu ir mažesniu dalyvavimu reabilitacijoje.

**Išvados:**

Orai veikia kaip kontekstinis modifikatorius, o ne pagrindinis OA simptomų determinantas. Meteorologinių veiksnių svarbos pripažinimas gali padėti pacientų konsultavimui, sezoniniam reabilitacijos planavimui ir baigtinių tyrimų dizainui.

## Methods

### Search Strategy

A structured literature search was conducted in *PubMed, ScienceDirect*, and *Google Scholar* databases with the objective to identify relevant studies published between January 2010 and May 2024. The search strategy followed predefined inclusion and exclusion criteria to ensure methodological consistency. The following keywords and Boolean operators were applied: (“osteoarthritis” OR “OA”) AND (“weather” OR “climate” OR “temperature” OR “humidity” OR “barometric pressure” OR “precipitation” OR “meteorological factors” OR “seasonal variation” OR “pain” OR “physical activity” OR “stiffness”). Searches were restricted to peer-reviewed human studies written in English. Reference lists of relevant papers were manually screened to identify additional studies. Duplicate entries were removed prior to screening.

### Eligibility Criteria

Studies were eligible for inclusion if they met *all* of the following criteria:

1. Included participants with clinically or radiographically confirmed osteoarthritis of any joint;
2. Examined at least one meteorological or seasonal exposure variable – specifically, temperature, humidity, barometric pressure, precipitation, wind speed, or diurnal temperature range – measured by official meteorological sources;
3. Reported quantitative outcomes related to pain, stiffness, functional limitation, physical activity, or healthcare utilization;
4. Provided sufficient methodological detail describing the study design, sample, exposure measurement, and analysis.

The exclusion criteria in use comprised: non-human or in vitro studies; conference abstracts, editorials, or narrative reviews without primary data; and interventional studies employing artificial heat or cold exposure.

### Screening and Selection

All retrieved titles and abstracts were independently screened by two reviewers against the eligibility criteria. Full texts of potentially relevant articles were then assessed in duplicate. Disagreements were resolved through discussion until consensus was reached. After duplicate removal and full-text evaluation, 15 studies satisfied inclusion criteria and were included in the qualitative synthesis.

### Data Extraction

For each included study, data were extracted on the publication year and country, study design and sample size, joint site affected, meteorological exposures and data sources, measured outcomes, statistical methods, main findings, and funding as well as conflicts of interest. Extraction was performed independently by both reviewers to ensure consistency.

Risk of bias was qualitatively appraised across domains of participant selection, exposure measurement, confounding control, and outcome assessment. The overall methodological quality among primary observational studies was considered to present low to moderate risk of bias, with transparent analytical procedures and clearly reported results.

### Classification of Included Studies

Fifteen studies published between 2010 and May 2024 met the final inclusion criteria. Of these, 10 were primary observational investigations directly analyzing meteorological influences on OA outcomes, and 5 served as contextual or epidemiologic sources providing a background on OA pathophysiology, management, and the global burden.

The primary OA–weather studies were: Brennan 2012 (*Int Orthop*); Queiroga 2013 (*Curr Orthop Pract*); Dorleijn 2014 (*Pain*); Timmermans 2014 (*BMC Musculoskelet Disord*); Timmermans 2015 (*J Rheumatol*); Timmermans 2016 (*J Phys Act Health*); Ferreira 2016 (*Osteoarthritis Cartilage*); Fu 2020 (*Scand J Rheumatol*); Wu 2022 (*BMC Musculoskelet Disord*); and Iconaru 2024 (*J Clin Med*).

**Table 1:** Characteristics of the 10 primary observational studies included in the review

First Author, Year	Country/Region	Study Design	Sample Size	Joint Site	Key Meteorological Variables	Main Outcomes	Key Findings
**Brennan, 2012**	Ireland	Short-term observational	53	Hip (end-stage)	Temperature, barometric pressure	Pain severity (VAS)	Pain ↑ with absolute change in barometric pressure
**Queiroga, 2013**	Brazil	28-day observational	40	Hand	Temperature, humidity, barometric pressure	Pain, stiffness, function	Cold & humidity strongly correlated with symptoms
**Dorleijn, 2014**	Netherlands	2-year prospective cohort	222	Hip	Temperature, humidity, barometric pressure, precipitation	WOMAC pain & function	Small associations (≤1% variance)
**Timmermans, 2014**	6 European countries (EPOSA)	Cross-sectional	2,519	Various	Self-perceived weather sensitivity	Joint pain (NRS)	67% weather-sensitive, higher pain scores
**Timmermans, 2015**	6 European countries (EPOSA)	Cross-sectional	2,458	Various	Temperature, humidity, barometric pressure	Joint pain	Humidity × cold interaction
**Timmermans, 2016**	6 European countries (EPOSA)	Cross-sectional	2,439	Various	Temperature, humidity, precipitation	Outdoor physical activity	Temperature ↑ PA; humidity ↓ PA
**Ferreira, 2016**	Australia	Case-crossover	354	Knee	Temperature, humidity, barometric pressure, precipitation	Risk of pain exacerbation	No strong associations
**Fu, 2020**	Australia	Case-crossover	252	Hip	Temperature fluctuations	Risk of pain exacerbation	>20 °C fluctuation → OR 3.89
**Wu, 2022**	Taiwan	Case-crossover	8,130	Various	Temperature, humidity, precipitation	Physical therapy utilization	Temperature ↑ utilization
**Iconaru, 2024**	Romania	Seasonal observational	104	Knee	Seasonal variation (winter vs summer)	Pain, stiffness, ROM	Winter ↑ pain & stiffness

*Note:* The following abbreviations were used: PA = physical activity; ROM = range of motion; ↑ = increased; OR = odds ratio; NRS = numeric rating scale; VAS = visual analogue scale; WOMAC = Western Ontario and McMaster Universities Osteoarthritis Index.

These encompassed case-crossover, cohort, and cross-sectional designs conducted across Europe, Asia, Australia, and South America, with sample sizes ranging from 28 to 8 130 participants.

Whereas, the contextual and review sources included: Bannuru 2019 (*Osteoarthritis & Cartilage*, OARSI guidelines); Wilkinson 2020 (*Calcified Tissue Int*.); Jang 2021 (*Int J Mol Sci*); GBD 2023 (*Lancet Rheumatology*); and Wang 2023 (*Ann Med*). These sources were used to contextualize epidemiologic, mechanistic, and management insights but were not pooled quantitatively.

### Analytical Overview

Primary studies used diverse analytical frameworks – most commonly conditional logistic regression, multilevel mixed models, or linear regression – to examine relationships between meteorological variables and OA outcomes. Exposures included temperature, relative humidity, barometric pressure, precipitation, and diurnal temperature range with time-windows typically spanning 24 to 72 hours. Outcomes most frequently assessed pain intensity (VAS or WOMAC), stiffness, function, or physical activity.

Meteorological data were generally obtained from national weather agencies such as the Bureau of Meteorology (Australia), Royal Netherlands Meteorological Institute, EMBRAPA (Brazil), Taiwan Weather Bureau, and EPOSA European dataset. Analyses routinely adjusted for potential confounders including age, sex, body-mass index, and psychological factors.

### Quality and Bias Considerations

All included primary studies were peer-reviewed and methodologically transparent. The most common limitations were reliance on self-reported pain, restricted climatic variability within a single-country samples, and incomplete adjustment for psychosocial confounders. These factors were acknowledged by the original authors. Risk of bias was qualitatively judged as low to moderate, and contextual reviews were used only to reinforce interpretation of epidemiological trends rather than combined in quantitative synthesis.

### Summary

This methodological process yielded a final dataset of 15 peer-reviewed articles (published in 2010–2024), including 10 primary observational studies and 5 contextual analyses encompassing diverse climates and populations. Together, these studies provide the empirical foundation for evaluating how temperature, humidity, barometric pressure, and related meteorological factors influence osteoarthritis symptoms, physical activity patterns, and healthcare utilization worldwide.

## Introduction

Osteoarthritis (OA) is a chronic degenerative joint disease involving progressive cartilage loss, subchondral bone remodeling, and mild synovial inflammation. It is the most common form of arthritis and a leading cause of pain and disability, most often affecting the knees, hips, and spine. The condition develops through a combination of mechanical stress, low-grade inflammation, and genetic susceptibility. Repeated joint loading activates chondrocytes to release matrix-degrading enzymes such as matrix metalloproteinases and ADAMTS proteases, which weaken the cartilage structure [1]. Cytokines including interleukin-1β, tumor necrosis factor-α, and prostaglandin E_2_ amplify this process, maintaining inflammation and promoting tissue damage. The infrapatellar fat pad contributes through secretion of adipokines and cytokines, while subchondral bone remodeling encourages vascular and nerve ingrowth, linking structural change to chronic pain [1].

Genetic variants in signaling pathways such as GDF5, BMP, Wnt, and IHH influence joint morphology and cartilage homeostasis, predisposing individuals to earlier degeneration [2]. Environmental influences may act on these same mechanisms: fluctuations in temperature, humidity, and barometric pressure can alter synovial fluid viscosity, vascular tone, and neural excitability, potentially enhancing pain in sensitized joints [3], [4], [5]. Activation of TRP ion channels, particularly TRPA1, may explain cold- and pressure-related pain responses [4]. Together, these processes show that OA pain arises from both structural and environmental influences, providing biological plausibility for weather-related symptom variability.

Osteoarthritis is one of the most common and disabling musculoskeletal conditions worldwide. It affects an estimated 595 million people, or 7.6% of the global population, according to the 2020 Global Burden of Disease study – which denotes an increase of over 130% since 1990 [6]. The knee is most frequently affected, followed by the hand and hip, and prevalence rises sharply with age; women are consistently affected more than men [6]. Regional variation is marked, with the highest rates in high-income Asia-Pacific and North America and the lowest in Southeast Asia and sub-Saharan Africa [6]. Beyond personal suffering, OA imposes a major socioeconomic impact through healthcare costs, productivity loss, and long-term disability – for instance, estimated in the United States to have risen from $128 billion in 2003 to $304 billion in 2013 [2].

OA primarily affects older adults and women, but obesity, prior joint injury, and genetic predisposition also contribute to onset and progression [1], [2], [6]. Mechanical overload not only drives cartilage damage and pain but may also heighten sensitivity to external environmental stressors such as barometric or temperature change. These interactions between intrinsic and external influences make OA an ideal condition for studying how biomechanical and environmental factors combine to shape symptom patterns and disease burden.

Clinically, OA presents with joint pain, stiffness, crepitus, and loss of mobility, most often in the knees, hips, or hands [1]. Pain typically fluctuates during the day and does not always correlate with radiographic findings; many patients with mild structural damage report substantial symptoms. This discrepancy highlights that OA pain reflects both structural and non-structural mechanisms, and that external factors – such as the weather or circadian rhythms – may influence pain perception independently of cartilage loss. Symptom variability, therefore, results from the combined effects of mechanical load, inflammation, and environmental modulation [1].

Current management aims to relieve pain, improve the function, and slow down progression. The 2019 OARSI guidelines recommend a non-surgical, patient-centered approach centered on education, exercise, and weight control [7]. Regular physical activity – such as walking, strengthening, or tai chi – reduces pain and supports mobility. Topical NSAIDs are preferred for knee OA, while oral NSAIDs may be used cautiously. Intra-articular corticosteroids or hyaluronic acid injections can provide short-term relief, and joint replacement remains the most effective option in advanced disease. Environmental factors, including temperature and activity setting, may also affect symptoms and treatment outcomes, providing context for exploring how weather influences OA management [7].

Osteoarthritis is a leading cause of disability and a reduced quality of life among older adults. In 2020, the global age-standardized years-lived-with-disability rate reached 255 per 100,000 – which represents a 9.5% increase since 1990 [6]. OA ranked seventh among the causes of disability in people aged 70 years and older and 14^th^ across all ages. The burden is projected to rise further, with knee OA cases expected to increase by 75%, hip OA by 79%, and other sites nearly doubling by 2050, bringing the total global prevalence close to one billion individuals [6]. Obesity accounts for about 20% of this burden, while joint injury, occupational strain, and metabolic inflammation add to progression. These trends highlight OA as a growing public-health challenge requiring prevention, early management, and equitable access to rehabilitation and surgery [6].

The disease often begins long before symptoms appear. Structural abnormalities such as developmental dysplasia of the hip, Legg–Calvé–Perthes disease, and femoroacetabular impingement create uneven load distribution that accelerates cartilage wear [2]. Even minor deformities can lead to early damage. With aging, repetitive loading, and inflammation, degeneration becomes gradual and irreversible. Reduced mobility fosters muscle weakness and comorbidities that accelerate decline, showing that OA progression reflects developmental, mechanical, metabolic, and environmental influences rather than aging alone [2].

Weather has long been recognized as influencing human physiology and well-being. Modern research supports that temperature, humidity, and barometric pressure alter tissue elasticity, perfusion, and inflammatory activity – which are processes directly relevant to joint mechanics [8]. In OA, these physiological effects provide a basis for patients’ reports that weather changes aggravate symptoms. Understanding of these interactions offers important context for exploring how external climatic variation contributes to daily fluctuations in OA pain and function.

Across populations, cold, humid, and rapidly changing weather are the most frequently reported aggravating conditions [3], [9], [10], [11]. In the EPOSA study, over two-thirds of older adults with OA identified themselves as weather-sensitive and reported higher pain scores than those who did not [10]. Humidity and temperature appeared to interact, with pain typically worsening under cold and damp conditions [9]. Short-term changes in temperature or barometric pressure may trigger transient pain responses, especially in advanced disease [3], [5]. These fluctuations can modify synovial-fluid viscosity, vascular tone, and mechanoreceptor sensitivity, thereby amplifying nociceptive signaling [8]. Although effects are small at the individual level, meta-analytic data confirm measurable associations between weather and OA pain, thus supporting the concept that meteorological variability contributes to symptom inconsistency [3], [4], [5], [8], [9], [10], [11], [12].

The perception that joint pain varies with weather is widespread. In EPOSA, nearly two-thirds of the participants reported weather-related symptom changes [10]. Those who described themselves as weather-sensitive reported higher pain levels even after adjustment for demographic and psychological factors [10]. Cold and damp conditions were most often cited, aligning with long-standing cultural beliefs [9], [10]. Regional differences suggest that behavior and climate adaptation influence how symptoms are perceived, with southern European participants reporting more pain than northern counterparts [9]. Psychological factors also contribute: female sex and higher anxiety independently predicted weather sensitivity [10]. Although patient perception does not always match objective data, it remains clinically important, shaping analgesic use, activity patterns, and interpretation of patient-reported outcomes [9], [11].

Physical activity is a cornerstone of OA management but is strongly influenced by weather. Large European studies show that older adults, particularly those with OA, are less active during cold, rainy, or humid conditions [13]. These environmental barriers reduce walking and gardening which are common forms of moderate exercise – leading to stiffness and pain. Favorable weather promotes outdoor activity, while prolonged adverse conditions encourage sedentary behavior and functional decline. Recognizing the role of climate in shaping activity patterns underscores the importance of adaptive rehabilitation strategies such as indoor and weather-independent exercise programs [13].

Despite the broad patient belief that weather affects OA symptoms, scientific evidence remains inconsistent. Some studies identify modest associations between temperature, humidity, or barometric pressure and pain, while others find none at all [3], [4], [5], [9], [12]. Meta-analytic results show small effect sizes, suggesting that weather explains only a minor fraction of symptom variability. Most research is observational and based on regional data, thus limiting precision, while psychological factors and attentional bias may amplify perceived effects [9], [12]. Overall, weather appears to act as a secondary modulator rather than a primary driver of OA pain. Future studies should integrate high-resolution meteorological data with daily symptom tracking and psychological profiling to better clarify causal pathways and distinguish true environmental influences from perception-based responses [3], [4], [5], [9], [12].

## Results

### Temperature and Symptom Associations

A comprehensive meta-analysis has shown a moderate inverse association between environmental temperature and osteoarthritis (OA) pain levels (*r* = -0.36, 95% CI -0.54 to -0.16), suggesting that colder conditions tend to aggravate pain across various climatic zones and joint types [10]. The analysis reported negligible heterogeneity (I^2^ = 0%), reinforcing the consistency of this relationship across the included studies.

Case-crossover investigations provide parallel evidence. In one study involving 252 Australian patients with hip OA, a temperature fluctuation greater than 20 °C within 72 hours was linked to a fourfold increase in pain exacerbations (OR 3.89, 95% CI 1.04–14.48; *p* trend = 0.04). In contrast, neither the highest nor the lowest daily temperatures predicted flare-ups independently [3]. Another analysis conducted in knee OA revealed that daily maximum temperatures above 30 °C were associated with roughly twofold risk of a pain flare (OR 2.18, 95% CI 1.01–4.74), though the effect lacked a clear dose–response trend [4]. Conversely, a 28-day observational study of advanced hip OA and a large EPOSA cohort found no independent link between the mean temperature and pain after accounting for humidity and barometric pressure [5], [14]. Interestingly, these datasets did identify an interaction effect, showing that low temperatures can amplify humidity-related pain (*p* = 0.01).

Regional and cohort evidence aligns with these patterns. In a Brazilian sample of patients with hand OA, daily temperature shifts were significantly correlated with pain, stiffness, and function in 40–88% of the individuals involved, with multivariate models explaining up to 88% of the variance (R^2^ = 0.52–0.88). This indicates that cold exposure consistently intensifies functional limitations [11]. Comparable results were reported in the EPOSA cross-sectional dataset of 712 older Europeans, where cold weather was the most commonly reported trigger (30%), and weather-sensitive individuals experienced higher mean pain scores than those who were not (4.1 ± 2.4 vs 3.1 ± 2.4, *p* <0.001) [9]. By contrast, a Dutch two-year follow-up of hip OA showed no significant relationship between temperature and either WOMAC pain or function (*p* = 0.84 and *p* = 0.61), implying that long-term acclimatization may dampen thermal effects [12].

Seasonal comparisons support this conclusion. Among Romanian adults with knee OA, pain levels were notably higher in winter than in summer (*p* <0.001, η^2^ = 0.432). Warmer seasons were also associated with greater joint flexibility, particularly in the evening (Δ = +6.6°, *p* = 0.014), illustrating that both thermal and diurnal rhythms modulate joint performance [8].

Environmental temperature also influences behavior. Within the broader EPOSA population of 2,439 older adults, each 1 °C rise in the average temperature corresponded to approximately 1.5 additional minutes of outdoor physical activity per day (*p* <0.001). This association was substantially weaker in participants with OA than in those without (B = 0.48 vs 1.98) [13].

In summary, the evidence indicates that colder climates and wide day-to-day temperature variations are linked to greater OA pain, stiffness, and functional decline, whereas warmer, more stable thermal conditions promote an improved mobility and symptom relief.

### Humidity and Symptom Associations

Overall, the available evidence points to a weak but consistent positive link between relative humidity and osteoarthritis (OA) pain severity. A meta-analysis synthesizing 14 observational studies reported a small yet statistically significant correlation (*r* = 0.086, 95% CI −0.05–0.22), suggesting that pain intensity tends to increase slightly under more humid conditions across different climates and joint types [9]. Similar trends were identified in the large EPOSA dataset, where both daily and 3-day mean humidity levels were positively associated with reported joint pain (B = 0.004, 95% CI 0.002–0.007; *p* <0.01). The relationship was strongest at lower temperatures (≤16.9 °C), thus indicating that humidity amplifies pain particularly in cold environments [14].

Findings from regional longitudinal studies complement these results. In a two-year Dutch cohort of hip OA, each 10% rise in humidity corresponded to roughly a one-point increase in WOMAC pain scores (95% CI 0.0–0.2; *p* = 0.02), though this variable explained less than 1% of the overall variance, reflecting a minor clinical effect [12]. Similarly, a Brazilian investigation focusing on hand OA demonstrated that humidity levels correlated with pain and stiffness changes in 39–85% of patients. Even after statistical adjustment, humidity remained a significant predictor of both pain and function (R^2^ = 0.49–0.85), though individual responses varied substantially [11].

Conversely, several controlled case-crossover studies did not confirm these associations. Analyses of Australian hip and knee OA cohorts found no statistically significant differences in pain flare risk across humidity categories (<59%, 59–79%, >79%), with odds ratios ranging from 0.68 to 1.25 (*p* >0.05) [3], [4]. These null results may be attributable to limited variability in climatic exposure within single geographic regions.

Self-reported and behavioral findings lend further support to the role of humidity in worsening symptoms. Within the EPOSA cross-sectional sample, damp or rainy weather was identified as the most common pain-aggravating factor, cited by 39% of participants. Individuals who perceived themselves as weather-sensitive reported significantly greater mean pain scores than those who did not (4.1 ± 2.2 vs 3.2 ± 2.1; *p* <0.001) [9]. Moreover, data from 2,439 older adults across Europe revealed that higher humidity was associated with reduced outdoor physical activity (B = -0.77, *p* <0.001), with this effect being most pronounced in Italy and the Netherlands [13].

In summary, the collective evidence suggests that high relative humidity, especially when coupled with cold air temperatures, modestly worsens OA-related pain, stiffness, and functional impairment. While the overall magnitude of this effect is small, the findings support a physiological basis involving increased synovial fluid viscosity and periarticular tissue swelling under cool, moist atmospheric conditions [8].

### Barometric Pressure and Symptom Associations

Research examining the effect of barometric pressure on osteoarthritis (OA) pain presents mixed findings. A quantitative meta-analysis reported a moderate positive correlation between atmospheric pressure and pain intensity (*r* = 0.35, 95% CI 0.15–0.53), with the strongest effects observed in hip and knee OA [10]. Among the fourteen included studies, eight identified a statistically significant relationship between a higher pressure and increased pain, and this pattern remained evident after adjusting for humidity and temperature, thus implying that pressure may exert independent influence on the symptom severity.

Observational evidence, however, has been less uniform. In the EPOSA cohort, elevated three-day mean barometric pressure showed a slight inverse relationship with pain (B = -0.005, 95% CI -0.009 to -0.001; *p* = 0.05), though this association weakened following full adjustment for confounders [14]. By contrast, a longitudinal Dutch study found a small but significant decline in functional outcomes with each 10-hPa increase in pressure (*p* = 0.02), explaining less than 1% of the overall variance and thus offering limited clinical relevance [12]. A short-term investigation of patients with advanced hip OA reached a different conclusion, showing that day-to-day pressure changes, rather than absolute pressure values, were more predictive of pain. Each 1-mm Hg fluctuation was associated with roughly a 2% rise in pain probability (β = -0.024, *p* = 0.005) [5].

Smaller regional studies provide additional context. In a Brazilian hand-OA cohort, pressure variability accounted for 40–60% of symptom fluctuations and remained an independent predictor alongside temperature (R^2^ = 0.52–0.88) [11]. Conversely, two Australian case-crossover analyses detected no linear or nonlinear relationships across pressure categories up to 1,024 hPa (*p* trend = 0.84) [3], [4]. Likewise, a Romanian seasonal study did not demonstrate a direct association between pressure and knee pain, though the authors proposed that atmospheric shifts might influence joint lubrication and stiffness indirectly [8].

Taken together, these studies imply that short-term barometric fluctuations, rather than sustained high or low pressure, are more likely to exacerbate pain in OA. Although the magnitude of this effect appears modest and varies by region and disease site, consistent signals from studies capturing daily atmospheric variability suggest a possible mechanosensory or fluid-dynamic mechanism operating within subchondral bone and synovial tissues.

### Other Meteorological Factors

Across the reviewed literature, precipitation, wind velocity, and sunlight exposure showed minimal or inconsistent links with osteoarthritis (OA) symptoms. The available meta-analysis highlighted that precipitation was seldom evaluated and, when it was, the reported outcomes varied widely without a clear directional trend [10]. Case-crossover studies from Australia similarly demonstrated no significant association between rainfall and the likelihood of pain flares, with odds ratios remaining near unity across rainfall categories (OR 0.96–1.35; *p* >0.05) [3], [4]. Longitudinal evidence from the Netherlands supported these findings: in a two-year cohort, short-term rainfall had no discernible effect on WOMAC pain or function. However, a two-day lag analysis suggested a marginal improvement in functional scores during wetter periods (estimate -0.2, 95% CI -0.3 to 0.0; *p* = 0.02), a pattern possibly reflecting reduced outdoor activity or recall bias [12]. The EPOSA dataset also reported a minor inverse relationship between daily rainfall and pain intensity (B = -0.006; *p* = 0.03), thereby indicating that rain corresponded with slightly lower pain perception, although the overall impact was trivial [14]. Similarly, an observational study of end-stage hip OA detected no measurable effect of rainfall or temperature on daily pain levels [5].

Research examining wind and sunlight exposure produced comparable results. In the Dutch cohort, neither average wind speed nor hours of sunshine were meaningfully related to pain or function (*p* >0.2 for both), and broader EPOSA data confirmed the lack of wind effects across six distinct European climates [12], [13], [14]. A single Turkish investigation reported a weak negative correlation between sunshine hours and pain intensity (*r* = -0.24), but this finding has not been replicated in subsequent studies [10]. Likewise, analyses from Brazil and Romania found no statistical associations between low rainfall, wind activity, or pain severity [8], [11].

Regional comparisons provide some evidence of climatic adaptation. Within EPOSA, participants living in warmer and drier climates such as Spain had the highest mean pain scores (≈5.4), while those in colder, wetter environments like Sweden reported the lowest (≈2.7). This pattern suggests that long-term acclimatization to local weather conditions may mitigate short-term meteorological effects [9]. Moreover, diurnal variation appeared more relevant than external weather factors: stiffness and reduced range of motion were consistently greater in the morning compared with the evening (*p* <0.001, η^2^ = 0.768), independent of seasonal differences [8].

Taken together, these findings imply that precipitation, sunlight, and wind exert little direct physiological influence on OA symptoms. Instead, behavioral and chronobiological mechanisms – such as daily activity timing, habitual routines, and long-term climatic adaptation – may play a more substantial role in shaping how patients experience pain and stiffness across different environments.

### Weather and Physical Activity in OA

Weather conditions appear to influence physical activity (PA), a crucial element for maintaining joint lubrication, mobility, and overall symptom control in osteoarthritis (OA). Data from the European Project on OSteoArthritis (EPOSA), which included 2,439 adults aged 65–85 years from six European countries, showed that 703 participants (29.6%) met ACR criteria for clinical OA. On average, those with OA spent less time outdoors compared with individuals without the disease (median 42.9 vs 51.4 minutes per day; *p* <0.01). When comparing specific activities such as walking, cycling, and gardening, no significant differences were observed, suggesting that the lower overall activity among OA participants reflected reduced duration or frequency, rather than selective avoidance of particular tasks [13].

Meteorological factors were also linked to differences in outdoor activity. For every 1 °C rise in the mean temperature, participants accumulated an additional 1.52 minutes of outdoor PA per day (B = 1.52; *p* <0.001). This association was considerably stronger among those without OA (B = 1.98; *p* <0.001) than in participants with OA (B = 0.48; *p* = 0.47), implying that joint limitations reduce responsiveness to favorable weather conditions [13]. Conversely, relative humidity was negatively associated with activity levels (B = -0.77; *p* <0.001), with the greatest declines observed in Italy (B = -2.82; *p* <0.001) and the Netherlands (B = -2.16; *p* <0.001). Walking time specifically fell as humidity increased (B = -0.34; *p* = 0.02), and a significant OA × humidity interaction showed that this reduction was more pronounced among individuals without OA (B = -0.46; *p* = 0.01) compared to those with OA (B = -0.03; *p* = 0.88).

Analysis of specific activity types revealed additional patterns. Gardening time increased with higher temperature (B = 1.23; *p* = 0.02) but decreased with both precipitation (B = -2.31; *p* = 0.03) and humidity (B = -1.07; *p* <0.01). Cycling did not show consistent associations with weather, and neither wind speed nor atmospheric pressure demonstrated significant effects on the total outdoor PA [13].

Population-based data from East Asia align with these observations. In a nationwide Taiwanese case-crossover study of 8,130 adults with OA (National Health Insurance Database, 2000–2013), the daily maximum temperature was significantly associated with an increased physical therapy utilization (OR 1.07, 95% CI 1.04–1.10; *p* <0.01), corresponding to a 7% rise in participation per 1 °C increment [15]. This effect was evident during cooler months (<23 °C) but plateaued at higher temperatures (>23 °C). Humidity showed a dual influence – p enhancing therapy use during hot weather (OR 1.05; *p* <0.01) but reducing it during colder periods (OR 0.97; *p* <0.01). Precipitation, however, consistently lowered the participation rates (OR 0.95; *p* <0.01). These findings suggest that weather patterns not only affect comfort and pain but also shape behavioral engagement in activity and rehabilitation.

Certain limitations temper these conclusions. Many studies relied on self-reported activity data and averaged weather exposures over multi-week periods, thus reducing sensitivity to short-term changes. Moreover, indoor activity was not systematically accounted for, which may have underestimated the total movement on days with poor weather. Clinically, the evidence underscores that cold, humid, and rainy conditions can deter outdoor activity, potentially accelerating functional decline. Since physical activity remains a key therapeutic strategy in OA management, encouraging indoor or weather-independent exercise options is essential to maintain joint health and mobility [13], [15].

### Limitations of Current Evidence

Across the reviewed literature, several methodological strengths enhance the credibility of the observed links between meteorological factors and osteoarthritis (OA) symptoms. The repeated use of case-crossover designs helped minimize between-person confounding by controlling for stable individual characteristics such as age, body mass index, and comorbidities [3], [4], [5]. Many investigations also incorporated objective weather data directly matched with time-stamped or daily self-reports of pain, improving temporal precision and reducing recall bias [3], [4], [5], [11], [12]. Large, multicenter cohorts – most notably, the European Project on OsteoArthritis (EPOSA) – added further robustness through harmonized diagnostic criteria, high response rates, and representation of diverse climatic regions [9], [13], [14]. Some studies expanded the analytical scope beyond pain to include functional and stiffness outcomes, by using real-time or seasonal measurements to capture fluctuations within individuals [8], [11], [12]. In addition, the inclusion of a quantitative meta-analysis provided a standardized synthesis across continents and methodologies, reinforcing the broader generalizability of associations related to temperature, humidity, and barometric pressure [10].

Despite these strengths, important limitations temper the interpretation of results. A considerable portion of the available data depends on self-reported pain or perceived weather sensitivity, both of which are vulnerable to expectancy bias and psychological influence [9], [11]. Few studies distinguished between indoor and outdoor exposure or accounted for the timing of physical activity, making it difficult to precisely link environmental conditions with joint symptoms [12], [14]. Most participant samples were older, predominantly female, and Caucasian, which restricts extrapolation to younger or more ethnically and geographically diverse populations [3], [4]. Many cohorts were small, region-specific samples exposed to relatively mild climatic variation, resulting in wide confidence intervals and limited ability to detect effects of extreme weather [5], [8], [11]. Even in larger datasets, meteorological variables were often averaged over 24-hour or multi-week periods, potentially masking short-term fluctuations relevant to symptom dynamics [12], [13]. Furthermore, the cross-sectional nature of several analyses prevents firm causal inference, while the majority of the observed relationships exhibited small effect sizes (ES <0.1, or <1% explained variance), thus suggesting limited clinical impact despite statistical significance [12], [13], [14].

In summary, although the overall evidence base benefits from robust design features and objective exposure linkage, its conclusions remain qualified by demographic homogeneity, reliance on subjective symptom measures, and incomplete characterization of behavioral and environmental moderators that likely influence individual sensitivity to weather changes.

Taken together, the evidence indicates that meteorological conditions exert a measurable yet modest effect on both osteoarthritis symptoms and activity patterns. Among all examined variables, temperature consistently showed the strongest influence – as colder and more variable conditions were associated with increased pain, stiffness, and functional limitation, whereas warmer and more stable environments tended to promote mobility and comfort. Humidity demonstrated a weaker but recurring connection to symptom worsening, particularly under cool and damp weather, while fluctuations in barometric pressure, rather than absolute pressure levels, appeared to provoke short-term pain exacerbations. In contrast, factors such as rainfall, sunlight exposure, and the wind speed yielded inconsistent or negligible associations, implying a more indirect role in symptom variability. Behavioral data further revealed that unfavorable weather reduces outdoor physical activity and therapy participation, which may exacerbate pain through diminished joint motion and muscle conditioning. Overall, although several statistically significant patterns emerged, the generally small effect sizes suggest that weather acts as a secondary modifier rather than a primary driver of OA symptoms. Collectively, these findings establish a foundation for the upcoming discussion, which explores the underlying physiological mechanisms, contextual influences, and clinical implications of weather-related symptom modulation.

## Discussion

### Principal Findings

This narrative review synthesizes recent evidence on the relationship between natural meteorological variation and osteoarthritis (OA) symptoms and physical activity [10]. Across diverse climates and study designs, temperature emerged as the meteorological factor with the most consistent association: colder and more variable conditions were linked to increased pain and stiffness, while warmer and stable temperatures favored mobility and symptom relief. Relative humidity showed a weaker but recurrent positive association with pain, particularly when combined with low temperatures. Barometric pressure influenced symptoms mainly through short-term fluctuations rather than absolute levels. Precipitation, wind speed, and sunlight exposure demonstrated minimal or inconsistent direct effects. Adverse weather was also associated with reduced outdoor physical activity and lower participation in rehabilitation programmes, suggesting an important behavioral pathway [13].

### Physiological and Mechanical Mechanisms

These patterns have plausible biological explanations. Lower temperatures increase synovial-fluid viscosity and periarticular muscle tone, thereby reducing joint lubrication and the range of motion. Rapid barometric pressure changes can alter the intra-articular–external pressure gradient, stretching the joint capsule and activating mechanoreceptors. High humidity may promote soft-tissue swelling, which is an effect amplified in cold conditions [8]. Collectively, meteorological factors appear to modulate – rather than independently drive – existing nociceptive and inflammatory processes within osteoarthritic joints [1].

### Subjective and Psychological Weather Sensitivity

A substantial proportion of patients (30–70%) perceive themselves as weather-sensitive, and these individuals consistently report higher pain levels [9]. Such perception is shaped by both physiological changes in sensory thresholds and psychological factors, including expectancy bias and cultural beliefs. This highlights that weather sensitivity in OA is a biopsychosocial phenomenon that influences symptom reporting and healthcare-seeking behavior [9].

### Behavioral and Environmental Interactions

Weather exerts indirect effects by shaping daily activity patterns. Unfavorable conditions reduce outdoor walking, gardening, and rehabilitation attendance, which may exacerbate stiffness and deconditioning over time. Conversely, warmer weather encourages physical activity, although patients with OA appear less responsive to favorable conditions than individuals without the disease. These behavioral responses underscore the importance of adaptive management strategies [13].

### Geographical and Cultural Context

Regional differences suggest long-term climatic adaptation. Individuals living in climates with stable weather patterns may experience less pronounced short-term effects, while cultural attitudes toward pain reporting and indoor versus outdoor lifestyles further modify the observed associations. Such contextual factors should be considered when generalizing findings across populations [9].

### Clinical and Research Implications

Clinicians can use these insights to counsel patients about expected symptom fluctuations and to recommend indoor exercise alternatives during cold or humid periods [7]. In research, meteorological variability should be recorded and adjusted for (or at least acknowledged) when interpreting patient-reported outcomes, especially in multicenter or seasonal trials. Future studies would benefit from wearable sensors for continuous activity and microclimate monitoring, objective biomarkers, and broader geographic representation.

## Conclusion

This review indicates that meteorological conditions have a measurable, though limited, impact on osteoarthritis (OA) symptoms and physical activity. Among the examined weather variables, temperature emerged as the most consistent factor: colder and less stable conditions were repeatedly linked to greater pain and stiffness, while warmer and more stable climates tended to support joint comfort and mobility. Humidity and barometric pressure fluctuations also contributed modestly, particularly under cool and damp conditions, whereas precipitation, sunlight exposure, and wind speed showed minimal or inconsistent relationships with symptom variation. Behavioral evidence complemented these findings, suggesting that unfavorable weather can reduce outdoor physical activity and participation in rehabilitation, indirectly worsening functional decline.

Although these effects reached statistical significance, their overall magnitude remained small, implying that weather functions as a contextual modifier rather than a primary determinant of OA symptoms. Recognizing this interaction carries both clinical and research relevance: patients can be advised on adaptive strategies to maintain activity levels during adverse weather, while investigators should account for meteorological variability when designing or interpreting outcome measures. Future research incorporating objective assessments, broader climatic representation, and longitudinal data will be essential to refine understanding of how weather patterns influence symptom dynamics and functional outcomes in OA.
